# High risk of disordered eating is associated with body composition, behavioural factors, and perceived stress among university students: a cross-sectional study from the UAE

**DOI:** 10.1017/jns.2026.10089

**Published:** 2026-04-15

**Authors:** Leila Cheikh Ismail, MoezAlIslam Faris, Dana N. Abdelrahim, Falak Zeb, Maysm N. Mohamad, Enas Amer, Maram Alali, Marianna Draghmeh, Najat Ben-Mustafa, Lily Stojanovska, Ayesha Salem Al Dhaheri

**Affiliations:** 1 Department of Clinical Nutrition and Dietetics, University of Sharjah, United Arab Emirates; 2 Nuffield Department of Women’s & Reproductive Health, University of Oxford, UK; 3 Department of Clinical Nutrition and Dietetics, Applied Science Private University, Jordan; 4 Research Institute for Medical and Health Sciences, University of Sharjah, United Arab Emirates; 5 Department of Nutrition and Health, United Arab Emirates University, United Arab Emirates; 6 Institute for Health and Sport, Victoria University, Australia

**Keywords:** Anthropometry, Body composition, Diet quality, Disordered eating, Eating attitudes, Perceived stress, University students

## Abstract

Disordered eating (DE) significantly affects both physical and mental health, contributing to morbidity, mortality, and considerable global healthcare costs. This cross-sectional study assessed the prevalence of high-risk DE and examined its associations with body composition, behavioural factors, diet quality, and perceived stress among university students in the United Arab Emirates. A total of 911 students were recruited using non-probability quota sampling (50.49% female). Body composition was measured using a TANITA BC-420MA body composition monitor. Usual dietary intake was assessed via a validated 65-item food frequency questionnaire. DE risk was assessed using the Eating Attitudes Test (EAT-26) and perceived stress using the PSS-10. Analysis included linear regression and independent-samples *t*-test (*p* < 0.05). High-risk DE (EAT-26 ≥ 20) prevalence was 30.3%. High-risk DE was significantly associated with higher body fat percentage (*β* = 0.121, *p* < 0.001), fat mass (*β* = 0.148, *p* < 0.001), fat-free mass (*β* = 0.079, *p* = 0.017), lean mass (*β* = 0.08, *p* = 0.016), total body water (*β* = 0.084, *p* = 0.011), and lower total body water percentage (*β* = −0.131, *p* < 0.001). High-risk students also reported higher intakes of fibre (*β* = 0.12, *p* = 0.018), beta-carotene (*β* = 0.14, *p* = 0.025), vitamin A (*β* = 0.13, *p* = 0.034), B12 (*β* = 0.15, *p* = 0.043), folate (*β* = 0.16, *p* = 0.006), and vitamin D (*β* = 0.16, *p* = 0.036). Compared with the low-risk group, high-risk DE was associated with higher adiposity markers and slightly higher perceived stress, and differed in selected nutrient intakes; sociodemographic characteristics were largely similar between groups except for smoking status. These findings support the implementation of targeted prevention strategies, including nutrition education, routine screening, and culturally tailored programmes, for young adults in the UAE.

## Introduction

Globally, the prevalence of disordered eating (DE) syndromes, including anorexia and bulimia nervosa, binge-eating disorders, and avoidant restrictive food intake disorder, are increasing and affecting 5.5–17.9% and 0.6–2.4% of young females and males, respectively,^(1)^ also known to be common among university students.^(2,3)^ A study in Ajman showed that the prevalence risk of eating disorders was 36.2% among university students^(4)^ while another study reported 13.9% among female university students in the UAE.^(5)^ Dieting, influence of media, peer pressure, obesity, and family pressure are the most significant sociocultural determinants of eating disorder reported in the UAE.^(4)^ Although factors such as media influence, dieting, and peer or family pressure are known to contribute to DE, they were not included in this study to maintain focus on measurable behavioural, anthropometric, dietary, and stress-related variables; these psychosocial factors will be considered in future research. A cross-cultural study among seven Arab countries demonstrated that the risk of DE attitudes among obese adolescent was two to three times higher than non-obese peers.^(6)^ Concerning the determinants of eating disorders, overweight, fat mass percentage, and waist to hip ratio were significantly associated with DE attitudes among Mediterranean adolescents.^(7)^

DE has significant impacts on physical and mental health and are an important cause of morbidity and mortality, as well as a substantial area of health spending globally. DE leads to a host of health impacts; these vary based on the type of disease, with anorexia having the most significant morbidity and mortality, causing bone weakness, anaemia, hypotension, organ failure, and in extreme cases, fatality. However, generally long-term outcomes with treatment is promising.^(8)^ In the U.S, the total direct economic cost of eating disorders was estimated to be 64.7 billion USD, with an additional decrease in individual well-being leading to an extra 326 billion USD of indirect economic impact.^(9)^ Despite its significant impacts, there are still considerable knowledge gaps in its prevalence and associated outcomes, particularly among young adults in non-Western countries, including the UAE and the broader Arab region. Eating disorders are caused by a strong interaction between sociocultural, psychological, and biological drivers, with prevalence, management, and outcome dependent on the person and place.^(10,11)^ Despite this, little is known about the prevalence of these conditions outside the Western world. In the UAE, globalization and social media, alongside economic prosperity, diverse food availability, mental health stigma, and high academic/professional pressure, may reinforce body-ideal norms and contribute to DE behaviours.^(12)^

Historically, in Arab countries, the prevalence of eating disorders has been presumed to be low due to a reduced cultural emphasis on low body weight, traditional norms often value fuller body shapes as a sign of health and prosperity, which may influence attitudes toward weight, diet, and body image.^(13)^ However, given the rapid evolution of many Arabic nations following industrialization, this is likely to change, as Western diets, media, and population have a cultural influence. However, to date, little research has been conducted to explore this phenomenon. This is likely to influence both incidences and outcomes in understudied regions and, subsequently, will require tailored health policy responses to ensure optimal population health in the area. Therefore, in this study, we aimed to identify the prevalence of high-risk eating disorders among young adults in the United Arab Emirates (UAE), as well as to identify associations with anthropometric, nutritional, and socioeconomic variables.

## Methods

### Study design

This manuscript reports a cross-sectional study investigating the associations between the risk of DE, anthropometry, diet quality, and perceived stress among university students in the UAE. The inclusion criteria were students enrolled at the University of Sharjah during the academic year 2018/2019 and those between 18 and 30 years old. Students under 18 years old, who were pregnant, have any implanted medical device, or suffer from any chronic disease that could affect their usual dietary and lifestyle habits were excluded. This study was conducted according to the guidelines laid down in the Declaration of Helsinki and all procedures involving human subjects were approved by the research ethics committee at the University of Sharjah (Reference number: REC-20-05-26-02-S). Written informed consent was obtained from all subjects. A pilot test was conducted with a small group of students to assess the clarity, reliability, and feasibility of the questionnaires and anthropometric measurement procedures before the main data collection.

### Participants

Eligible participants were approached on campus by the research team, who explained the study objectives and procedures and provided an information sheet. Students who verbally agreed to participate were asked to sign a written informed consent form. Anthropometric measurements were taken following standardized protocols, after which participants completed the validated food frequency questionnaire (FFQ), EAT-26, and PSS-10 questionnaires under researcher supervision. All measurements and questionnaire responses were collected during a single session to ensure consistency and minimize missing data.

Non-probability quota sampling was used to recruit participants from the University of Sharjah’s four geographically distinct campuses, ensuring representation across all main campuses. Eligible students were approached directly by the research team, provided with study information, and asked to provide written consent.

After applying the inclusion and exclusion criteria, a total of 911 students completed all study procedures and were included in the final analysis.

The minimum sample size was calculated based on Cochran’s formula, with a confidence interval of 95%:

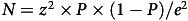




Where *z* = 1.96; P = (estimated proportion of the population that presents the characteristic) = 0.5; e (margin of error) = 0.05; N (sample size) = 384 participants, plus 20% (attrition rate) = 460 participants. Eligible participants were approached by the researchers, who explained the study’s aims and protocol and provided the students with an information sheet. Verbally consenting students were asked to sign a written consent form.

No prior screening was performed beyond the inclusion and exclusion criteria. All measurements and questionnaires were administered by trained research team members, who ensured standardized procedures and assisted participants as needed.

### Data collection tools

Independent variables were selected based on prior research identifying anthropometric, dietary, behavioural, and stress-related factors as relevant contributors to DE, and on the feasibility of reliably measuring these variables in our study population.

### Anthropometric assessment and body composition

Height measurements were taken using the SECA 769-Digital column scale (Seca Deutschland, Hamburg, Germany). Each participant was instructed to stand erect, with the back of the head, shoulder blades, buttocks, calves, and heels in constant contact with the stadiometer’s vertical height rod and their head oriented in the Frankfurt plane. The participant’s height was then measured by placing the horizontal headpiece on top of their head. Without shoes, height was measured twice to the nearest millimetre. The study used the average of the two values for each measurement. Several anthropometric parameters were then measured using the TANITA BC-420MA Body Composition Monitor (TANITA BC-420MA, Tanita Corp., Tokyo, Japan). Body weight, fat percentage, fat mass, fat-free mass, lean mass, total body water, total body water percentage, bone mass, basal metabolic rate, and body mass index were measured.

### Dietary assessment

Usual dietary intake was assessed using a self-reported, validated, reliable 65-item FFQ adapted from El Mesmoudi et al.^(14)^ The FFQ consisted of a list of foods with standard serving sizes, and participants were asked to indicate their usual consumption for each food item consumed in the previous year. The FFQ had nine possible responses: (1) never; (2) once per month; (3) once per week; (4) 2–4 times per week; (5) 5–6 times per week; (6) once per day; (7) 2–3 times per day; (8) 4–5 times per day; and (9) more than six times per day. The FFQ was converted into grams using household measurements, and an estimated average daily intake of dietary parameters was determined using dietary Processor Nutrition Analysis Software-ESHA (version 10.4; ESHA Research, Salem, OR, USA). The nutritional analysis covered a range of factors, including energy content, protein, carbohydrates, fibre, fat, saturated fat, monounsaturated fat (MUFA), polyunsaturated fat (PUFA), trans fat, cholesterol, Omega-3, and Omega-6. Essential vitamins such as A, beta-carotene, Thiamine, Riboflavin, Niacin, B6, B12, C, D, and E, along with folic acid, were included. Mineral components consisted of iron, magnesium, selenium, and zinc. Additionally, caffeine, a stimulant, was accounted for in the nutritional assessment. Dietary quality was assessed through a validated 65-item FFQ adapted from El Mesmoudi et al.^(14)^ Reported intakes were converted to grams and analysed using ESHA Nutrition Analysis Software to estimate daily nutrient intake. Although overall dietary adequacy scores were not calculated, this approach allowed comparison of macronutrient and micronutrient intake between participants with high- and low-risk DE.

### Eating attitudes

The eating attitude test (EAT-26) developed by Garner et al.^(15,16)^ was used to assess DE risks. The questionnaire contains 26 items, with response options scored as: always = 3, usually = 2, often = 1, and sometimes/rarely/never = 0, resulting in a total score range of 0–78.

### Perception of stress

The perceived stress scale (PSS-10) is widely used and validated as a psychological instrument for measuring the perception of stress.^(17,18)^ The PSS-10 scale includes ten items that inquire about thoughts and emotions from the previous month. In each item, respondents are asked how frequently they felt in a particular way, with the response options ranging from never (0) to very often (4). The total score can range from 0–40, with higher scores indicating higher perceived stress.

### Statistical analysis

All statistical analysis was performed using the R-based platform JASP (V0.18.1) and SPSS (Version 29). Univariate associations between continuous and ordinal variables were analysed through linear regression. Participants were categorized according to EAT-26 score, with scores ≥20 indicating high-risk DE and scores <20 indicating low-risk. These groups were evaluated for mean differences through independent-samples *t*-test. The alpha value (Type I error level) was set to *α* = 0.05, corresponding to a 95% confidence level. Therefore, *p*-values less than 0.05 are considered significant, with all *p*-values being two-tailed. Binary logistic regression was used to calculate odds ratios (OR) and 95% confidence intervals for the association between high-risk DE (EAT-26 score ≥ 20) and sociodemographic variables. Analyses examined associations between disordered-eating risk and (i) sociodemographic characteristics, (ii) perceived stress (PSS-10), (iii) anthropometric/body-composition measures, and (iv) dietary intake variables derived from the FFQ.

## Results

A total of 911 participants completed the study. There was a balance between males and females in the cohort (50.49% female), with the majority of participants being of Arabic background. The mean EAT-26 score in the cohort was 15.07, and the point prevalence of high risk for DE (EAT-26 score > 20) was 30.3%. The full descriptive data for the cohort is described in Table 1.

**Table 1.** Sociodemographic characteristics of the student cohort

Variable	Category	Mean (S.D.) n (%)
Age n (%)	18–21	697 (76.77)
	22–25	204 (22.39)
	26–30	10 (1.10)
Gender n (%)	Male	451 (49.51)
	Female	460 (50.49)
Nationality n (%)	UAE (citizen)	160 (17.56)
	Arab, GCC	259 (28.43)
	Arab, non-GCC	412 (45.23)
	Non-Arab	80 (8.78)
Place of living n (%)	Dorm	293 (32.16)
	External, family	594 (65.20)
	External, non-family	24 (2.63)
Smoker n (%)	Yes	218 (23.93)
	No	693 (76.07)
Income P.A. n (%)	<60,000AED	670 (73.55)
	60–100,000 AED	135 (14.82)
	>100,000 AED	106 (11.64)
College	Humanities	118 (13.0)
	Applied physics	340 (37.3)
	Medical and health sciences	453 (49.7)
Educational level	1st year	218 (23.9)
	2nd year	181 (19.9)
	3rd year	211 (23.2)
	4th year	246 (27.0)
	5th–6th years	55 (6.0)
Disordered eating risk n (%)	High risk (20+ EAT-26)	276 (30.30)
	Male	131 (29.05)
	Female	145 (31.52)
	Low risk (<20 EAT-26)	635 (69.70)
	Male	320 (70.95)
	Female	315 (68.48)
Years of higher education mean (SD)		2.73 (1.29)
BMI (kg/m^2^) mean (SD)		24.51 (5.03)
Body fat % mean (SD)		22.41 (9.64)
EAT26 score mean (SD)		15.07 (12.70)
PSS-10 mean (SD)		19.36 (5.74)

UAE, United Arab Emirates; GCC, Gulf Cooperation Council; AED, United Arab Emirates Dirham; SD, Standard deviation; Income P.A., Income per annum; BMI, Body mass index; EAT-26, Eating attitude test; PSS-10, Perceived Stress Scale. Data are presented as mean ± standard deviation for continuous variables and frequency (percentage) for categorical variables. Descriptive statistics were used.

High risk of eating disorder (EAT-26 > 20) was only associated with current smoking, with non-smokers significantly less likely to be at high risk (Odds Ratio [OR] = 0.696, *p* = 0.037). No other sociodemographic variables were associated with high risk (Supplementary Table 1). A high EAT-26 score was associated with significant increases in percent body fat (*r* = 0.121, *p* < 0.001), total fat mass (*r* = 0.148, *p* < 0.001), fat-free mass (*r* = 0.079, *p* = 0.017), lean mass (*r* = 0.08, *p* = 0.016), body water (*r* = 0.084, *p* = 0.011), bone mass (*r* = 0.084, *p* = 0.011), and basal metabolic rate (*r* = 0.084, *p* = 0.01), but a lower body water percentage (*r* = −0.131, *p* < 0.001) (Table 2).

**Table 2.** Associations between EAT-26 scores, anthropometric and densitometry variables

Variable	Correlation coefficient	*P* value
Body fat %	0.121	**<0.001**
Fat mass	0.148	**<0.001**
Fat-free mass	0.079	**0.017**
Lean mass	0.08	**0.016**
Total body water	0.084	**0.011**
Total body water %	−0.131	**<0.001**
Bone mass	0.084	**0.011**
Basal metabolic rate	0.085	**0.01**

Univariate linear regression analyses were performed, and results are presented as regression coefficients (r) with corresponding *p*-values. Bold indicates *p* < 0.05.

Individuals at high risk of eating disorders (EAT-26 score >20) scored an average of 0.884 points significantly higher on the PSS-10 (*p* = 0.033). In addition, those at high risk of eating disorders have higher mean values of FFM (27.41 ± 5.04 vs 27.14 ± 4.84, *p* = 0.28), lean mass (42.91 ± 7.23 vs 42.55 ± 7.02, *p* = 0.31), and TBW (33.36 ± 5.35 vs 33.07 ± 5.11, *p* = 0.29), although these differences were not statistically significant (Table 3). Also, individuals at high risk of eating disorders had higher mean BMR (1,476 ± 184 vs 1,462 ± 177 kcal/day, *p* = 0.21), FFM, lean mass, and TBW; however, these differences were not statistically significant. Statistically significant differences were observed only for body fat percentage (*p* < 0.001) and fat mass (*p* < 0.001). (Table 3).

**Table 3.** The difference in perceived stress, anthropometric, and densitometry variables between the high and low high and low risk of eating disorders

Variable	Low risk mean (SD)	High risk mean (SD)	Mean difference	*P* value
Perceived Stress Scale (PSS-10)	19.091 (5.932)	19.975 (5.222)	0.884	**0.033**
BMI (kg/m^2^)	24.02 (4.84)	25.62 (5.29)	1.6	**<0.001**
Body Fat %	21.629 (9.343)	24.192 (10.077)	2.6	**<0.001**
Fat mass	15.393 (9.628)	18.69 (11.387)	3.3	**<0.001**
FFM	53.242 (12.998)	54.928 (13.279)	1.69	0.074
Lean mass	50.52 (12.315)	52.123 (12.669)	1.6	0.074
TBW	37.69 (8.878)	38.893 (9.268)	1.2	0.065
TBW %	55.416 (6.393)	53.758 (6.614)	−1.7	**<0.001**
Bone mineral density	2.683 (0.608)	2.77 (0.62)	0.087	**0.049**

Group differences were assessed using independent-samples *t*-test. Data are presented as mean ± standard deviation. TBW, Total Body Water; FFM, Fat-Free Mass. Bold indicates *p* < 0.05.

Individuals who are at high risk of eating disorders have a greater overall intake of fibre (*p* = 0.018), with no other differences in macronutrient intake. Additionally, those at higher risk have significantly increased dietary intake of beta carotene (*p* = 0.025), Vitamin A (*p* = 0.034), vitamin B12 (*p* = 0.043), vitamin D (*p* = 0.036), and folic acid (*p* = 0.006). (Table 4).

**Table 4.** Difference in macro- and micro-nutrient intake between at high and low high and low risk of eating disorders

Nutrient	Low risk mean (S.D.)		High risk mean (S.D.)		*P* value
Energy (kcal.)	3658.112	(2165.35)	3742.732	(2143.829)	0.587
Protein (g)	127.675	(117.26)	126.276	(73.68)	0.855
Carbohydrate (g)	476.018	(304.249)	498.505	(297.508)	0.302
Fibre (g)	43.627	(34.471)	49.543	(35.199)	**0.018**
Fat (g)	153.433	(110.861)	150.562	(92.199)	0.706
Sat. Fat (g)	55.34	(47.484)	53.155	(32.492)	0.486
MUFA (g)	35.799	(23.949)	33.979	(23.37)	0.289
PUFA (g)	25.16	(18.634)	23.967	(16.851)	0.361
Trans fat (g)	3.699	(42.977)	0.944	(1.03)	0.287
Cholesterol (mg)	424.457	(423.429)	406.437	(322.367)	0.528
Omega-3 (g)	2.366	(3.601)	2.036	(1.506)	0.143
Omega-6 (g)	22.232	(17.251)	21.018	(15.12)	0.312
Vit. A (IU)	12543.73	(13173.86)	14646.03	(14864.66)	**0.034**
Beta-carotene (mcg)	4023.696	(4492.99)	4796.116	(5327.53)	**0.025**
Thiamine (B1) (mg)	2.673	(1.949)	2.891	(1.967)	0.123
Riboflavin (B2) (mg)	4.143	(5.563)	4.114	(2.948)	0.936
Niacin (B3) (mg)	37.442	(25.234)	38.481	(24.245)	0.563
Pyridoxine (B6) (mg)	29.763	(39.185)	30.968	(37.908)	0.667
Folate (B9) (mcg)	309.962	(644.539)	452.536	(876.617)	**0.006**
Cobalamin (B12) (mcg)	9.672	(16.003)	12.244	(20.795)	**0.043**
Vit. C (mg)	163.995	(220.246)	192.919	(280.746)	0.095
Vit. D (IU)	181.886	(226.22)	220.123	(302.855)	**0.036**
Vit. E (mg)	40.23	(121.399)	34.684	(52.602)	0.466
Iron (mg)	536.086	(919.792)	581.264	(852.973)	0.486
Magnesium (mg)	245.499	(287.983)	269.886	(317.686)	0.256
Selenium (mcg)	329.999	(629.615)	322.047	(325.04)	0.843
Zinc (mg)	73.534	(93.979)	76.738	(92.111)	0.643
Caffeine (mg)	71.78	(401.13)	62.106	(138.729)	0.696

Group differences were assessed using independent-samples *t*-test. Data are presented as mean ± standard deviation. Bold indicates *p* < 0.05.

## Discussion

Eating disorders are increasing in prevalence globally; however, due to the strong influence of sociocultural, economic, and demographic variables, there is a strong need for regional data to inform local decision-makers. In this study, we show that 30% of young adults surveyed are at high risk of eating disorders and that these individuals had poorer anthropometric indices of health but a higher intake of several beneficial macro- and micronutrients.

The prevalence of eating disorders in the Middle East and North Africa (MENA) region has been suggested to vary widely, with recent review findings that range from 2.8–75.8% in different countries.^(19)^ There is very little current data available from the UAE, with the most recent studies from 2013 and 2014 finding a range of 14.6–41.2%.^(20,21)^ The estimates found in our data broadly align with this, with a total estimate of 30.3% of the young adults surveyed having a high risk of eating disorders based on the EAT-26 questionnaire. Our finding is supported by a previous study conducted in Ajman, UAE, which showed 36.2% prevalence risk of eating disorder.^(4)^ It is important to note that this study included only female adolescents, whereas our study focused on both male and female university students in the UAE. In contrast, another study showed only 13.9% among university students in the UAE.^(5)^ The discrepancies in prevalence estimates between our study and prior UAE studies may be attributed to differences in sample size, age range, and study population characteristics. For instance, the study in Ajman^(4)^ included only female adolescents, whereas our study included both male and female university students aged 18–30, resulting in a broader and more balanced representation. Additionally, variations in recruitment strategies, campus settings, and regional dietary and cultural practices may contribute to differences in observed prevalence^(19,22)^. However, that was not shown in our study, with no associations between gender and risk of DE, with females and males showing largely similar prevalence of high-risk scores (29.05% [Male] vs 31.52% [Female]). The likely cause of this is unclear but may be due to changing sociocultural attitudes toward food and physical appearance, such as increased exposure to Western media promoting thin ideals, widespread use of social media emphasizing body image, greater availability of calorie-dense Western-style foods, and heightened academic and professional pressures, may contribute to DE behaviours among university students in the UAE. There was also a lack of other associations between the risk of DE and socioeconomic and demographic variables in the data set, including age, level of education, income, or ethnicity. However, it should be noted that the age range evaluated in this study was relatively small, given the recruitment amongst college students. Only smoking status was associated with a high risk of DE, a relationship that has been shown previously with bulimia nervosa and binge eating disorders, but not with anorexia nervosa.^(23)^ Given that the EAT-26 tool does not provide discrimination between types of eating disorders, the only risk of DE overall, it is unclear if this relationship is the case in the data shown in our study. The general lack of association with sociodemographic variables makes the information gained in this study more applicable at a population level rather than for risk group identification.

The relationship between DE and body composition is variable and dependent on the specific eating disorder. In our data, individuals at high risk of eating disorders had higher indices of adiposity and obesity than those at low risk. This agrees with previous research, in which increased rates of overweight and obesity have been shown in cohorts at high risk of DE.^(24)^ Similarly, the high risk of DE attitudes was significantly associated with fat mass, fat-free mass, and muscle mass among Kuwaiti college men^(25)^ and obesity among students in seven Arab countries.^(6)^ Moreover, it was also observed that high DE attitude was significantly associated with BMI among schoolgirls in the UAE.^(26)^ The causative agent in this relationship is unclear, however, as overweight and obesity, as well as body dissatisfaction, are likely causes of DE,^(27,28)^ although some eating disorders are known to lead to weight gain, most notably binge eating disorder.^(29)^ Despite the associations between overweight and obesity, in this cohort, the high risk of DE was not associated with increased energy consumption. However, there was a small mean difference between the two groups. This makes the cause of the increased adiposity in high-risk individuals challenging to identify, with factors such as stress, timing of food consumption, and alcohol intake potential mediators.

Interestingly, there was also a positive association between EAT-26 score and bone mineral density (BMD), and individuals at high risk had higher bone mass than those at lower risk. The relationship between eating disorders and bone mass is complex, with obesity broadly associated with increased BMD, while anorexia and underweight are associated with osteoporosis.^(30,31)^ These relationships are particularly important to understand given the age of the cohort, who were largely in the period of peak bone accrual, in which disruption leads to significant skeletal health impact.^(32)^ We acknowledge that although several associations between EAT-26 scores and body composition parameters were statistically significant, the correlation coefficients are generally low (|r| ≤ 0.15). This indicates that the strength of these relationships is weak, and their clinical or practical significance may be limited despite statistical significance, likely influenced by the large sample size (*n* = 911).

In recent years, the focus of nutrition research has moved from quantity (overnutrition) to nutrient intake patterns, which is a stronger indicator of overall health outcomes. While those at high risk of eating disorders had higher rates of overweight and obesity, they conversely had some indicators of a higher diet nutrient intake pattern. Those at high risk of DE had a greater intake of fibre, beta carotene, and vitamins A, B9, and B12, as well as vitamin D. The high intake of fibre by individuals with a high risk of DE attitude is supported by a previous study, which revealed that the odds of eating disorders was higher in adolescents who follow a high fibre dietary pattern.^(33)^ Moreover, previous studies demonstrated that the mean intake of major macro (carbohydrate, protein, and fat) and micronutrients (sodium, phosphorus, and thiamine) were high than the recommended amounts among adults with DE attitudes.^(34)^ In the present study, dietary data partly support this observation, as individuals at high risk of DE showed significantly higher intakes of fibre and selected micronutrients (beta-carotene, vitamins A, B12, D, and folic acid), while no significant differences were observed in total energy intake or most macronutrients.^(35–37)^ This finding is in contrast to other research, which has largely found high EAT-26 scores to be associated with poorer nutrient intake patterns through a range of indices.^(38,39)^ It is difficult to identify a likely cause for the differences between the dietary intakes of the two groups, with some potential mediators including greater vigilance over diet in high-risk individuals or potential influences of commonly eaten regional foods in the UAE. However, nutritional risk is associated with DE attitudes including nutrients imbalance, electrolytes disturbances, dehydration, and decrease in bone mass density.^(40,41)^ It was also reported that vitamin C and calcium intake was lower among adolescent girls and boys, respectively, with DE behaviours.^(42)^ Likewise, children and adolescents with avoidant/restrictive food intake disorder obtained only 20–30% of the recommended amounts for most vitamins and minerals, with significantly lower intake of vitamin B1, B2, C, K, zinc, iron, and potassium.^(43)^ In our study, individuals at high risk of DE also had significantly higher perceived stress scores (PSS-10), suggesting that elevated stress levels may contribute to or exacerbate DE behaviours among university students. This finding underscores the importance of incorporating stress management and mental health support into preventive and interventional strategies for this population.

While this study is the largest of its kind conducted in the UAE, incorporating a critical update on regional eating attitudes, there are some limitations to its generalizability. Firstly, we utilized the EAT-26 questionnaire as a proxy for DE risk. Although the EAT-26 is the most well-established tool for the assessment of DE risk, it does not provide a true indication of the prevalence of specific eating disorders as it is non-diagnostic. Given the significant variability in the management of the different subcategories of eating disorders, it is important to note that this only provides an assessment of the risk of DE. Additional studies on the prevalence of diagnosed eating disorders are required to give more insight into the specific burden of these diseases. However, it is important to note that the under-diagnosis of eating disorders presents significant challenges to this.

The second important consideration to the applicability of the findings in this study is the narrow age range of the participants. We deliberately recruited only younger adults, as this is the age range in which eating disorders are the most prevalent and impactful. However, it makes the generalizability of the findings somewhat limited to this population.

A third limitation is reliance on self-reported dietary intake assessed via FFQ. This questionnaire can introduce recall bias, lack of specificity in food choices, and misestimation of portion size. However, this could lead to underreporting and influence the dietary data due to cultural stigma. In such case, the findings of the study will be biased and reduce generalizability of the study.

Globally, the prevalence of eating disorders is increasing and have a significant impact on the psychological and physical health of younger people. However, in large parts of the world, there is little information available on these diseases. In this study, the largest of its kind in the UAE, we showed that 30.3% of young adults are at high risk of DE. Those individuals have increased indicators of adiposity, overweight, and obesity with fluctuated diet quality. This data should assist in informing policy responses to limit the impact of eating disorders both locally and globally, allowing a tailored approach to prevention. The policy makers and government should support the intervention programmes include mandatory nutrition education, routine health screening, awareness campaigns, culturally tailored community programmes and increase access to support services.

## Supporting information

10.1017/jns.2026.10089.sm001 Cheikh Ismail et al. supplementary material Cheikh Ismail et al. supplementary material

## Supplementary material

The supplementary material for this article can be found at https://doi.org/10.1017/jns.2026.10089.
