# Supplementary material for: High risk of disordered eating is associated with body composition, behavioural factors, and perceived stress among university students: a cross-sectional study from the UAE
Source: J Nutr Sci. 2026 Apr 15;15:e25. doi: 10.1017/jns.2026.10089 (PMC13126061; doi:10.1017/jns.2026.10089)
Supplement: Cheikh Ismail et al. supplementary material [file S2048679026100895sup001.docx]

Supplementary Table 1. Associations between sociodemographic variables and high-risk score on EAT-26.

| Variable | Estimate | Standard Error | Odds Ratio | p-value |
| --- | --- | --- | --- | --- |
| Age (22-25) | 0.225 | 0.207 | 1.253 | 0.275 |
| Age (26-30) | -0.004 | 0.74 | 0.996 | 0.995 |
| Nationality (Arab, GCC) | 0.283 | 0.222 | 1.326 | 0.203 |
| Nationality (Arab, Non-GCC) | -0.152 | 0.213 | 0.859 | 0.477 |
| Nationality (Non-Arab | -0.591 | 0.335 | 0.554 | 0.078 |
| Living Place (External, Family) | -0.085 | 0.162 | 0.919 | 0.6 |
| Living Place (External non-family) | -0.776 | 0.544 | 0.46 | 0.154 |
| Smoker (No) | -0.363 | 0.174 | 0.696 | **0.037** |
| Income (60-100k AED) | 0.122 | 0.209 | 1.129 | 0.56 |
| Income (>100k AED) | 0.317 | 0.229 | 1.373 | 0.165 |
| College (Humanities) | -0.313 | 0.234 | 0.731 | 0.18 |
| College (Applied Physics) | -0.405 | 0.232 | 0.667 | 0.081 |
| College (Medical and Health Sciences) | 16.713 | 1455.398 | 1.812×10+7 | 0.991 |
| Educational Level (1-2 years) | -0.027 | 0.225 | 0.974 | 0.905 |
| Educational Level (2-3 years) | -0.171 | 0.223 | 0.843 | 0.442 |
| Educational Level (3-4 years) | 0.082 | 0.222 | 1.085 | 0.712 |
| Educational Level (4-5 years) | -0.598 | 0.451 | 0.55 | 0.185 |
| Educational Level (5-6 years) | -0.587 | 0.705 | 0.556 | 0.404 |

Associations were examined using binary logistic regression analysis, and results are presented as odds ratios (ORs) with corresponding p-values. Bold indicates p < 0.05.
